# Transcription factor Tlx1 marks a subset of lymphoid tissue organizer-like mesenchymal progenitor cells in the neonatal spleen

**DOI:** 10.1038/s41598-019-56984-w

**Published:** 2019-12-31

**Authors:** Yuta Ueno, Keiko Fujisaki, Shoko Hosoda, Yusuke Amemiya, Shogo Okazaki, Chihiro Notsu, Chiharu Nishiyama, Yo Mabuchi, Yumi Matsuzaki, Akihisa Oda, Ryo Goitsuka

**Affiliations:** 1 Division of Development and Aging, Research Institute for Biomedical Sciences, Tokyo University of Science, Chiba, Japan; 2 Laboratory of Molecular Biology and Immunology, Department of Biological Science and Technology, Tokyo University of Science, Tokyo, Japan; 3 Department of Biochemistry and Biophysics, Graduate School of Medical and Dental Sciences, Tokyo Medical and Dental University, Tokyo, Japan; 4 Department of Life Sciences, Faculty of Medicine, Shimane University, Shimane, Japan; 5 Department of Pediatrics, Nara Medical University, Nara, Japan; 6 Imaging Frontier Center, Tokyo University of Science, Chiba, Japan

**Keywords:** Stem-cell research, Imaging the immune system

## Abstract

The spleen is comprised of spatially distinct compartments whose functions, such as immune responses and removal of aged red blood cells, are tightly controlled by the non-hematopoietic stromal cells that provide regionally-restricted signals to properly activate hematopoietic cells residing in each area. However, information regarding the ontogeny and relationships of the different stromal cell types remains limited. Here we have used *in vivo* lineage tracing analysis and *in vitro* mesenchymal stromal cell assays and found that Tlx1, a transcription factor essential for embryonic spleen organogenesis, marks neonatal stromal cells that are selectively localized in the spleen and retain mesenchymal progenitor potential to differentiate into mature follicular dendritic cells, fibroblastic reticular cells and marginal reticular cells. Furthermore, by establishing a novel three-dimensional cell culture system that enables maintenance of Tlx1-expressing cells *in vitro*, we discovered that signals from the lymphotoxin β receptor and TNF receptor promote differentiation of these cells to express MAdCAM-1, CCL19 and CXCL13, representative functional molecules expressed by different subsets of mature stromal cells in the spleen. Taken together, these findings indicate that mesenchymal progenitor cells expressing Tlx1 are a subset of lymphoid tissue organizer-like cells selectively found in the neonatal spleen.

## Introduction

The spleen is a secondary lymphoid organ that serves various important roles in the generation of immune responses against blood-borne pathogens and in extramedullary hematopoiesis, as well as acting as a filter to remove and process aged red blood cells^1–3^. These physiological functions rely on spatially distinct compartments, in which specific hematopoietic cell populations interact with unique stromal cell populations^4–9^. The white pulp (WP) is comprised of the T cell zone, called the periarteriolar lymphoid sheath (PALS) because it surrounds the central arterioles, and the B cell follicle, which is surrounded by the marginal zone, where distinct subsets of B cells and macrophages reside. Outside of the WP and marginal zone, the red pulp (RP) contains macrophage-rich splenic cords where blood is filtered. The stromal network of the T cell zone in the WP is formed by fibroblastic reticular cells (FRCs), which are characterized by the expression of podoplanin, and recruits T cells by secretion of chemokines such as CCL19 and CCL21. In contrast, follicular dendritic cells (FDCs) in the B cell follicles selectively express CD21/CD35 and CD16/32 and attract B cells by the expression of CXCL13. The marginal zone contains a specialized stromal cell population, the marginal reticular cells (MRCs), which express MAdCAM-1 as well as CXCL13, and line the marginal sinus located between the WP and RP. The RP is constructed of abundant venous sinusoids with heterogenous stromal cell populations, including ER-TR-7^+^ RP fibroblasts.

From the developmental viewpoint, the mouse spleen starts to develop as the dorsal splenopancreatic mesenchyme on embryonic day (E) 10–10.5, regulated by spatially overlapping and restricted expression of a set of transcription factors^10^. At E14.5, the mesenchyme specified to become the spleen is then vascularized, leading to the recruitment of hematopoietic cells, mainly myeloid- and erythroid-lineage progenitors, to become a perinatal hematopoietic site^11,12^. Some lymphoid-lineage cells, such as lymphoid tissue inducer-like (LTi) cells, appear in the late embryonic stage^13^. However, maturation of the structural architecture of the WP and marginal zone within the RP area only begins to occur during the neonatal period after birth^14^ via the interaction of B cells with splenic stromal cells. This interaction provides a lymphotoxin ββ receptor (LTβR)- as well as a TNF receptor (TNFR)-triggered noncanonical NFκB signal for differentiation of specialized stromal cells^15–18^, designated lymphoid tissue organizer (LTo) cells, into functional FRCs, FDCs and MRCs. By using genetic lineage tracing, a cell population expressing Nkx2-5, a transcription factor expressed in E10.5 dorsal splenopancreatic mesenchyme, was recently demonstrated to give rise to all of the postnatal spleen mesenchymal cells, including perivascular mural cells of the central arterioles^19^, identifying Nkx2-5^+^ cells as the origin of splenic LTo cells. However, information regarding the origin as well as the relationship of splenic mesenchymal stromal cells still remains limited.

Similar to Nkx2-5, Tlx1, also known as Hox11, is one of the transcription factors expressed in E10.5 dorsal splenopancreatic mesenchyme in a relatively restricted manner compared to other transcription factors. Probably as a result, its absence causes asplenia without detectable abnormalities in other organs^20,21^. In order to better understand the function/cell fate of Tlx1 expressing cells, we have previously generated a mouse strain harboring a mutant *Tlx1* gene allele in which *CreER* and *Venus* genes are knocked into the first exon of the *Tlx1* gene (*Tlx1*^*CreER-Venus*^)^22^. We demonstrated that Tlx1 is required for cell fate determination of mesenchymal cells of the spleen primordium, as Tlx1-deficient progeny in the embryonic spleen primordium became dorsal pancreatic mesenchymal cells. Furthermore, Tlx1 expression was found to persist in a specific subpopulation of mesenchymal cells in the perifollicular area of the postnatal spleen. Also, Tlx1^+^ cells, because they have the highest expression of CXCL12 among the splenic stromal cells, serve as an extramedullary hematopoietic stem cell niche for emergency hematopoiesis^23^. Mesenchymal progenitor cells are the major constituent of the hematopoietic niche in the bone marrow^24^. However, it remains unclear whether such mesenchymal progenitors with tri-lineage (adipogenic, osteogenic and chondrogenic) differentiation potential as well as hematopoietic niche activity exist in the neonatal spleen and, if so, whether these cells also function as LTo cells to differentiate into functionally mature stromal cells that support structural integrity of the developing spleen.

To clarify these issues, in the present study, we used both *in vivo* lineage tracing and a novel *in vitro* three-dimensional (3D) culture system to examine whether neonatal Tlx1-expressing cells function as mesenchymal progenitor cells with the potential to differentiate into the mature stromal cells that organize the structural and functional integrity of the spleen.

## Results

### Tlx1 marks stromal cells selectively localized in the neonatal spleen

**Figure 1:**
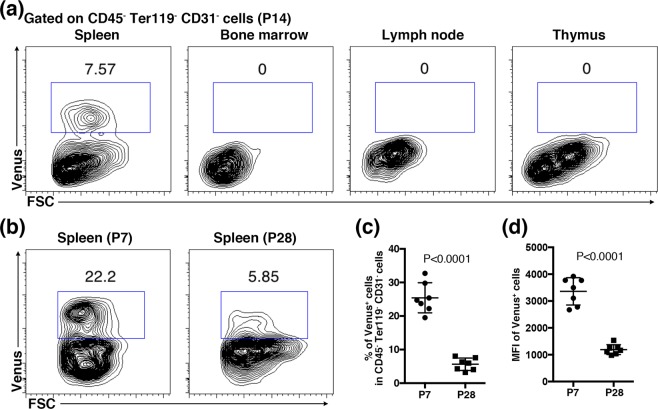
Tlx1 expression in stromal cells during the postnatal period. (**a**) Representative flow cytometric profiles of CD45^−^Ter119^−^ CD31^−^ stromal cells in the spleen, bone marrow, lymph node and thymus from *Tlx1*^*CreER-Venus*^ mice (P14). The gate used to identify the Venus^+^ cell population is outlined and numbers above outlined areas indicate percent events in each gate. A detailed gating strategy is provided in Fig. S1. (**b**) Representative flow cytometric profiles of Venus^+^ stromal cells in the spleen from *Tlx1*^*CreER-Venus*^ mice (P7 and P28). (**c**) Frequencies of Venus^+^ cells in CD45^−^Ter119^−^ CD31^−^ stromal cells in the spleen from *Tlx1*^*CreER-Venus*^ mice (P7 and P28). (mean ± SD, n = 7). (**d)** The MFI of Venus fluorescence in Venus^+^ cells from the spleen from *Tlx1*^*CreER-Venus*^ mice (P7 and P28). (mean ± SD, n = 7).

We first examined the tissue localization of Tlx1-expressing cells by using Venus expression as a marker in *Tlx1*^*CreER-Venus*^ heterozygous mice at postnatal day 14 (P14). Although no Venus expression was detected in the CD45^+^Ter119^+^ hematopoietic cell compartments (Fig. S1a), a population of CD45^−^Ter119^−^CD31^−^ stromal cells in the spleen was clearly positive (Fig. 1a). By contrast, such Venus^+^ stromal cells were not observed in the bone marrow, lymph node or thymus (Fig. 1a), indicating that Tlx1-expressing stromal cells are a unique cell population selectively present in the neonatal spleen. In addition, the frequency and mean fluorescence intensity (MFI) of Venus^+^ cells significantly decreased during postnatal development (from 25.43 ± 1.70% at P7 to 5.64 ± 0.70% at P28 and from 3359 ± 192 at P7 to 1191 ± 69 at P28, respectively) in the stromal cell compartment in the spleen (Fig. 1b-d).

**Figure 2:**
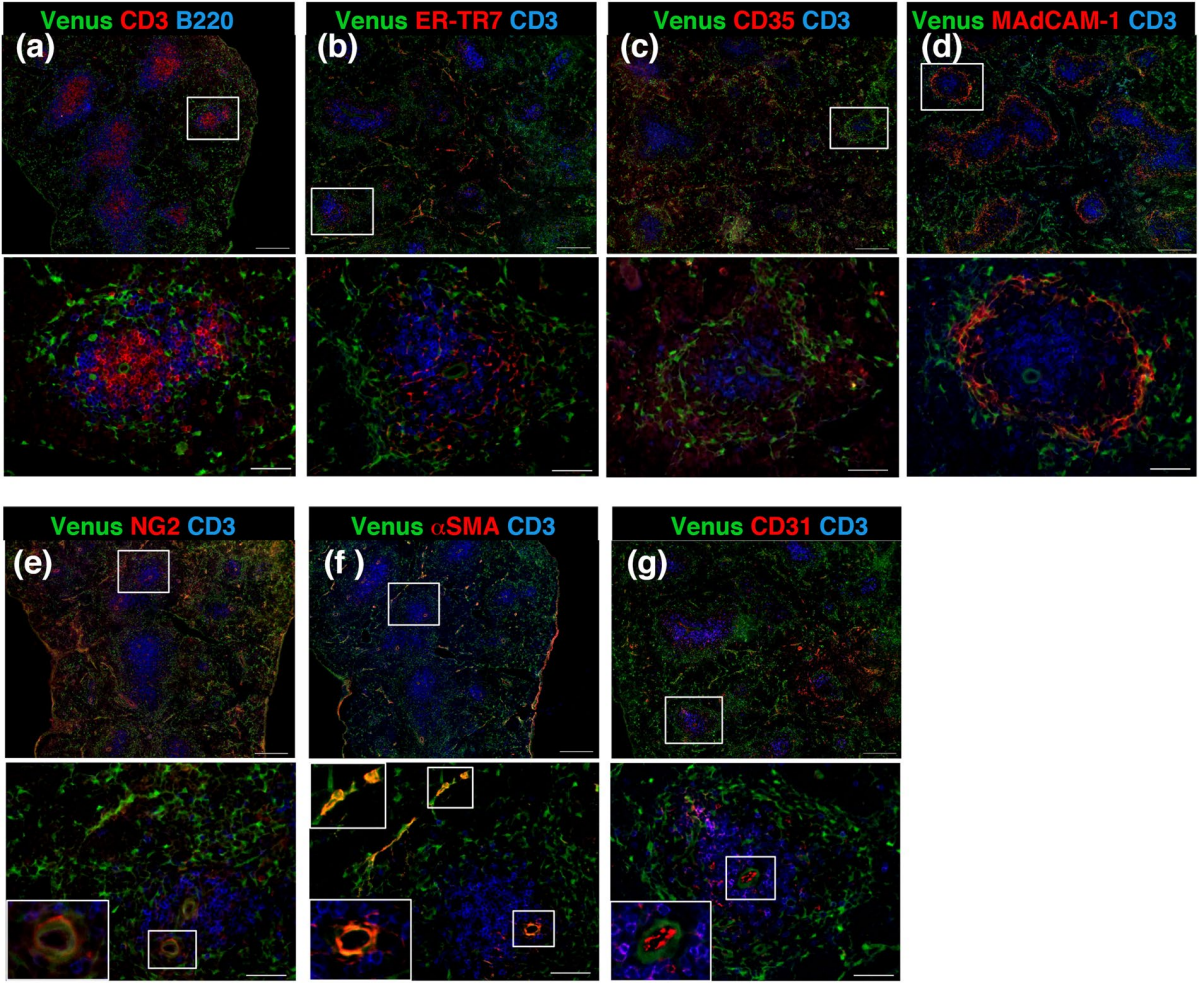
Tlx1 is expressed in red pulp stromal cells surrounding the follicles and in periaortic mural cells of the neonatal spleen. (**a**–**g**) Representative tissue section images of Venus^+^ cells in the spleen of *Tlx1*^*CreER-Venus*^ mice (P7). Tissue sections were stained with the indicated antibody combinations. Higher magnification images (lower panels) are indicated by an inserted rectangle in the upper images. Scale bars indicate 100 μm and 50 μm in upper and lower panels, respectively. (n = 5).

We next analyzed the distribution of Tlx1-expressing cells in the neonatal spleen (P7) by using antibodies to previously identified spleen stromal cell markers combined with anti-GFP antibody for detecting Venus expression. The majority of Venus^+^ cells were scattered throughout the red pulp, but with a tendency to surround follicles of the WP where CD3^+^ T cells and B220^+^ B cells reside (Fig. 2a). Venus expression did not overlap with ER-TR7 or CD35 (Fig. 2b,c), markers for FRCs or FDCs in the CD3^+^ T cell area and CD3^−^ non-T cell areas of the WP, respectively. However, although the majority did not, a few Venus^+^ cells closely attached to the follicles appeared to overlap with MAdCAM-1, a marker for MRCs lining the marginal sinus that separates the splenic WP and RP (Fig. 2d). In addition, Venus expression was observed in NG2^+^ *α*SMA^+^ mural cells that cover the endothelial cells of the central arterioles (Fig. 2e,f), although the CD31^+^ vascular endothelial cell itself did not express Venus (Fig. 2g). Thus, these findings suggest that Tlx1-expressing cells in the neonatal spleen are previously unknown stromal cells.

### Tlx1 marks stromal cells in the neonatal spleen that phenotypically resemble mesenchymal progenitors and lymphoid tissue organizer cells

**Figure 3:**
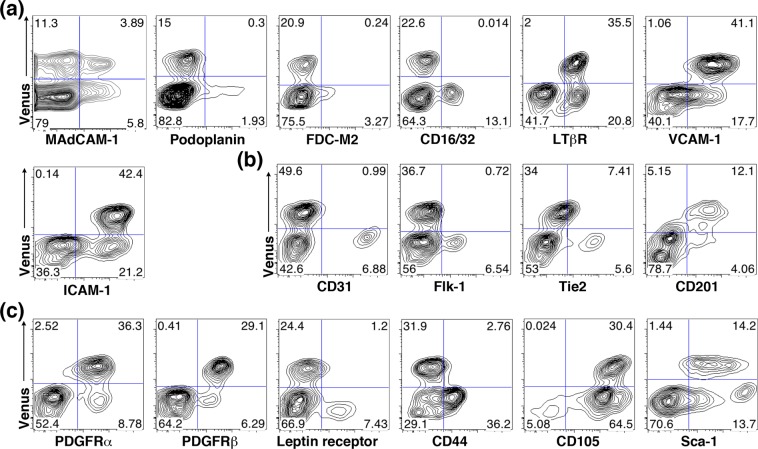
Tlx1 marks mesenchymal progenitor-like cells in the neonatal spleen. (**a**–**c**). Representative flow cytometric profiles of Venus^+^ stromal cells in the spleen of *Tlx1*^*CreER-Venus*^ mice (P7) stained for (**a**) mature and functional cell surface markers, (**b**) vascular endothelial markers and (**c**) mesenchymal progenitor cell markers. Staining with isotype-matched control antibodies and the use of control *Tlx1*^*−/−*^ spleen stromal cells was applied to determine the background fluorescence. Numbers in each quadrant indicate percentage of total cells analyzed. (n = 6).

To characterize the Tlx1-expressing stromal cells of the neonatal spleen (P7) in more detail, we examined cell surface markers on Venus^+^ cells by flow cytometry. Consistent with the immunohistochemical findings shown in Fig. 1, we found two Venus^+^ cell populations, with and without MAdCAM-1 expression, in addition to Venus^−^ MAdCAM-1^+^ cells (Fig. 3a). Furthermore, nearly all Venus^+^ cells were negative for podoplanin and FDC-M2 or CD16/32 (Fig. 3a), markers for FRCs and FDCs, respectively, but did express LTβR and high levels of VCAM-1 and ICAM-1. In this regard, they are phenotypically similar to lymphoid tissue organizer cells, which are important for the development of lymph nodes^25^. As for vascular endothelial markers, Venus^+^ cells were negative for CD31, Flk-1 (vascular endothelial growth factor receptor-2), and Tie2 (angiopoietin receptor 2), but positive for CD201 (endoglin receptor) (Fig. 3b). Furthermore, mesenchymal progenitor cell markers, including platelet-derived growth factor receptor (PDGFR *α* and β) and CD105 were expressed on Venus^+^ cells (Fig. 3c). Although mesenchymal progenitor cells in the bone marrow have been reported to express the leptin receptor^26^ and/or PDGFR*α* with Sca-1 (so called P*α*S cells)^27^, Venus^+^ cells in the neonatal spleen were leptin receptor-negative and Sca-1^dull−+^. Since mesenchymal progenitor cells of different tissues display similar but distinct phenotypes and functions^28^, these findings suggest that Tlx1-expressing cells are spleen-restricted mesenchymal progenitor cells with the potential to function as lymphoid tissue organizer cells.

### Neonatal Tlx1-expressing stromal cells have tri-lineage differentiation potential *in vitro*

**Figure 4:**
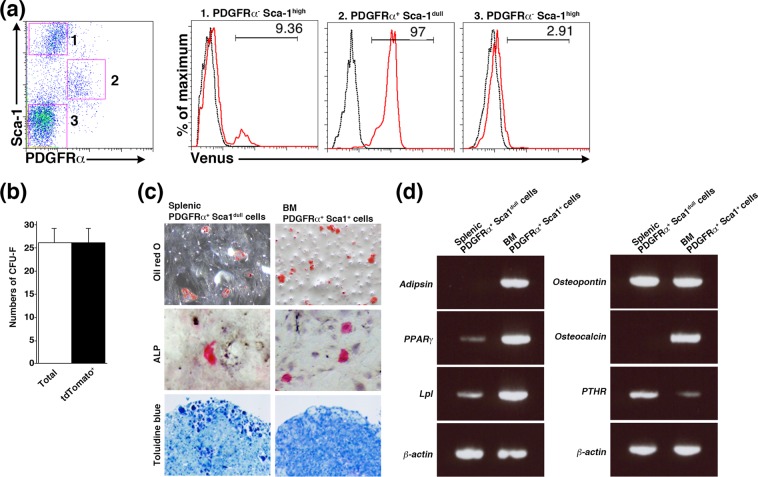
Tlx1-expressing cells contain a mesenchymal cell population with *in vitro* tri-lineage differentiation potential. (**a**) Representative flow cytometric histograms showing Venus expression in CD45^−^Ter119^−^ stromal cell compartments of the spleen from *Tlx1*^*CreER-Venus*^ mice (P7) (designated 1–3 in the left flow plot) stained with anti-PDGFR*α* and Sca-1 antibodies. Venus^+^ cells are enriched in PDGFR*α*^+^ Sca-1^dull^ stromal cell population. (**b**) Numbers of total and tdTomato^+^ CFU-Fs 7 days after plating of whole splenic stromal cells from tamoxifen-treated *Tlx1*^*CreER-Venus*^; *Rosa26*^*tdTomato*^ mice (P7). (**c**) Tri-lineage differentiation of the indicated cells from wild-type C57BL/6 mice (P7). Adipogenesis, osteogenesis and chondrogenesis were evaluated by oil red O, alkaline phosphatase (ALP) and toluidine blue staining, respectively. (n = 3). (**d**) RT-PCR analysis of transcription factors and lineage-specific genes. Expression of adipocyte- (*Adipsin*, *PPARγ*, and *Lpl*), and osteocyte-specific (*Osteopontin*, *Osteocalcin*, and *PTHR*) markers, 3 weeks after differentiation induction. Data are representative of three independent experiments.

To understand whether Tlx1-expressing cells in the neonatal spleen functionally behave as mesenchymal progenitor cells, we examined their potential to form colony-forming unit fibroblasts (CFU-F) and to undergo tri-lineage differentiation (adipogenic, osteogenic and chondrogenic) *in vitro*. In these experiments, instead of Venus^+^ cells from *Tlx1*^*CreER-Venus*^ mice, we used stromal cells from neonatal spleen of tamoxifen-treated *Tlx1*^*CreER-Venus*^; *Rosa26*^*tdTomato*^ mice for the CFU-F assay and PDGFR*α*^+^ Sca-1^dull^ stromal cells, which are enriched in Tlx1-expressing cells (Fig. 4a), from wild-type mice for the tri-lineage differentiation assay. We adopted this strategy because Venus fluorescence was reduced to undetectable levels immediately after conventional two-dimensional cell culture and the sorted Venus^+^ cells did not grow well *in vitro*. The reasons for this are unclear, but a potential reduction in Tlx1 protein levels in stromal cells from *Tlx1*^*CreER-Venus*^ mice due to haploinsufficiency might affect cell growth *in vitro*. For the CFU-F assay, we treated *Tlx1*^*CreER-Venus*^; *Rosa26*^*tdTomato*^ mice (P7) with tamoxifen to mark Tlx1-expressing cells as tdTomato^+^ and, one day after the treatment, the entire population of CD45^−^Ter119^−^CD31^−^ splenic stromal cells was plated in culture dishes. After one week, all the colonies formed were tdTomato^+^ (Fig. 4b), indicating that in the neonatal spleen cells with CFU-F ability expressed Tlx1. We next compared the tri-lineage differentiation potential of sorted splenic PDGFR*α*^+^ Sca-1^dull^ stromal cells enriched in Tlx1-expressing cells with bone marrow P*α*S cells under the same culture conditions. Freshly prepared cells were cultured in maintenance media for two passages, then in differentiation-induction media for an additional 2–3 weeks, according to a protocol described previously^27^. Like bone marrow P*α*S cells, splenic PDGFR*α*^+^ Sca-1^dull^ stromal cells showed adipogenic, osteogenic and chondrogenic differentiation, demonstrated by oil red O, alkaline phosphatase (ALP) and toluidine blue staining, respectively (Fig. 4c).

When we compared the expression of adipocyte- and bone-related genes between splenic PDGFR*α*^+^ Sca-1^dull^ cells and BM P*α*S cells, the splenic PDGFR*α*^+^ Sca-1^dull^ cells cultured under adipogenic differentiation conditions expressed profoundly lower levels of adipocyte-related genes, including *adipsin*, *peroxisome proliferator-activated receptor (PPAR)γ* and *lipoprotein lipase (Lpl)*, than did BM P*α*S cells (Fig. 4d, left), suggesting that the splenic PDGFR*α*^+^ Sca-1^dull^ cells had weaker adipogenic potential than the BM P*α*S cells. Furthermore, among the bone-related genes, *osteocalcin* expression was not detected in splenic PDGFR*α*^+^ Sca-1^dull^ cells cultured under osteogenic conditions (Fig. 4d, right). Thus, these findings indicate a limited *in vitro* tri-lineage differentiation capacity of splenic PDGFR*α*^+^ Sca-1^dull^ cells compared to BM P*α*S cells. Because the tri-lineage differentiation potential of splenic PDGFR*α*^+^ Sca-1^dull^ cells was not assessed at the clonal level, there remains the possibility that this cell fraction contains either bi-potential or uni-potential precursors for adipocytes, osteocytes and chondrocytes. Nevertheless, Tlx1-expressing cells in the neonatal spleen are likely to be the cell type that is capable of differentiation into the three lineages of mesenchymal cells *in vitro*, because all of the CFU-F capability in the neonatal spleen resides in Tlx1-expressing cells.

### Neonatal and young adult Tlx1-expressing cells have the potential to differentiate into mature stromal cells in the spleen

**Figure 5:**
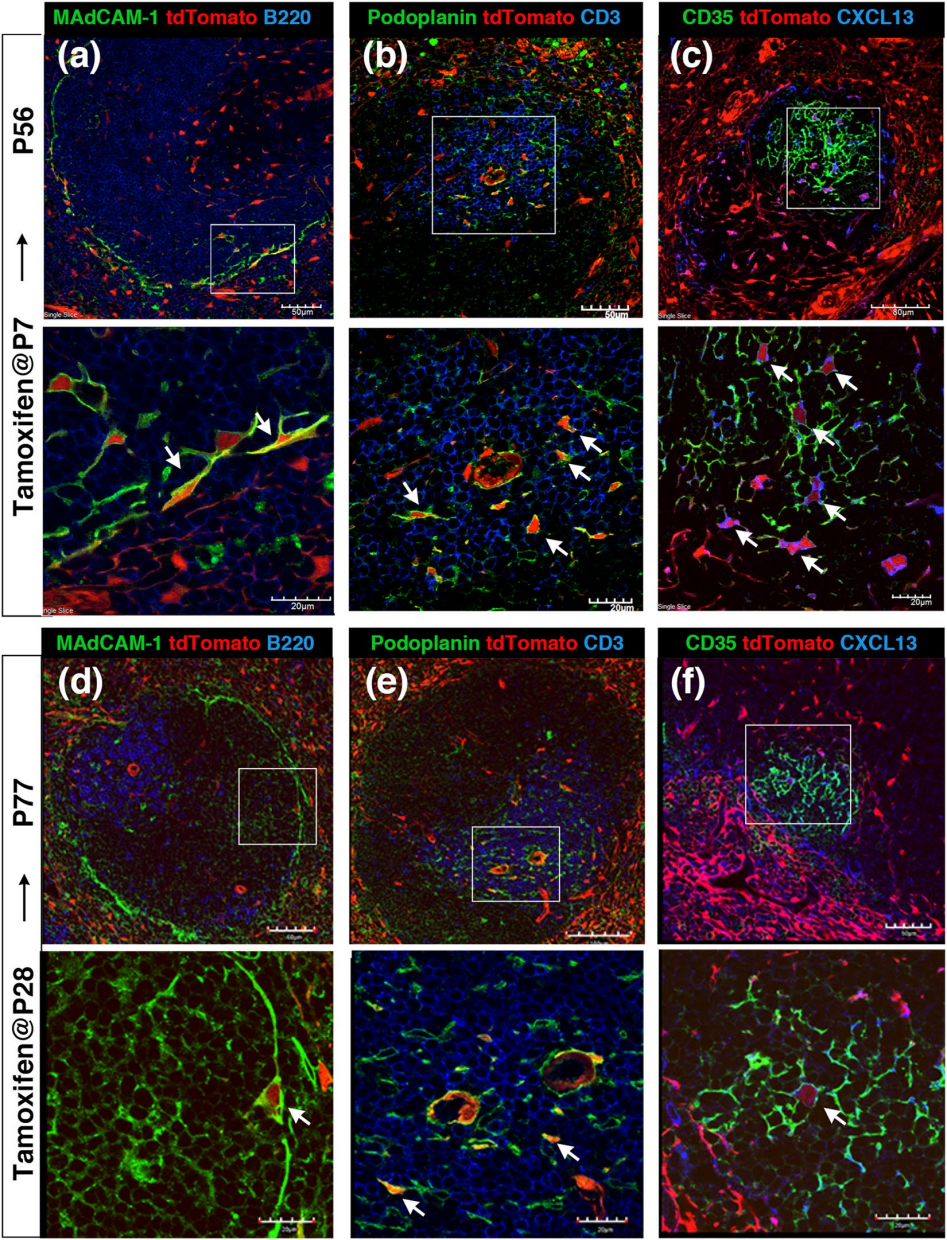
Differentiation potential of Tlx1-expressing cells into mature splenic stromal cells. (**a–f**) Representative tissue section images of the progeny of Tlx1-expressing cells in the spleen of *Tlx1*^*CreER-Venus*^; *Rosa26*^*tdTomato*^ mice. Tissue sections were stained with the indicated antibody combinations. Higher magnification images (lower panels) are indicated by an inserted rectangle in the upper images. Scale bars indicate 50 μm and 20 μm in upper and lower panels, respectively. **(a**–**c)** Mice were treated with tamoxifen at P7, and immunohistochemical analysis was carried out at P56 (n = 3). **(d**–**f)** Mice were treated with tamoxifen at P28, and immunohistochemical analysis was carried out at P77 (n = 3).

**Figure 6:**
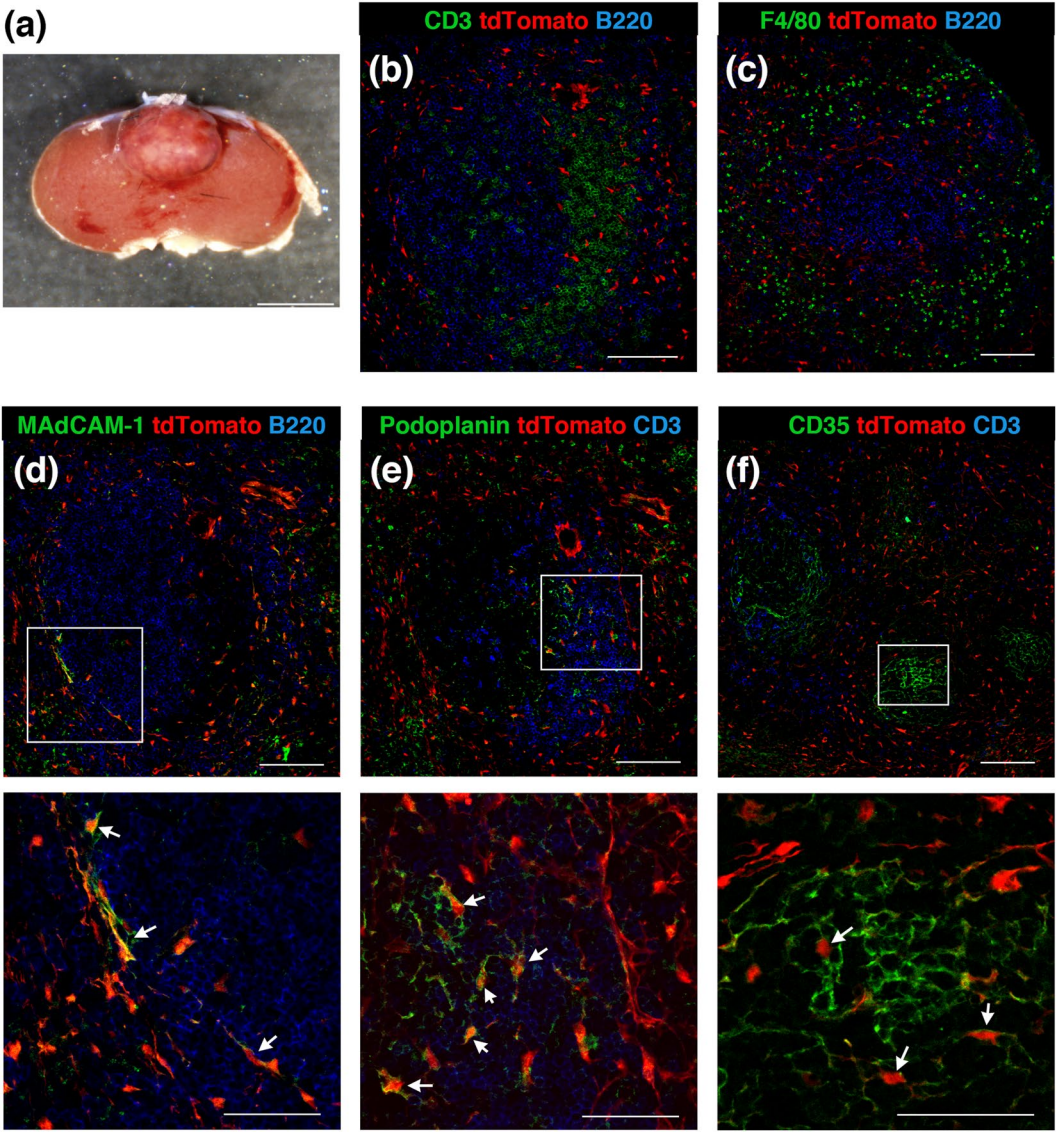
Spleen-resident Tlx1-expressing cells differentiate into mature stromal cells in the ectopically grafted spleen. (**a)** Macroscopic appearance of the transplanted spleen under the kidney capsule. *Tlx1*^*CreER-Venus*^; *Rosa26*^*tdTomato*^ embryos (E16.5) were labeled by tamoxifen administration into pregnant mice. The spleen was removed from embryos (E17.5) and then grafted under the kidney capsule of wild-type 8-week-old C57BL/6 mice. The transplanted spleen was analyzed by immunohistochemistry 4 weeks later. **(b**,**c)** Formation of structural architecture of the WP showing T and B cells (**b**) and of the red pulp resident macrophages (**c**) in the grafted spleen. Scale bars indicate 100 μm. (**d**–**f**) Representative tissue section images of the progeny of Tlx1-expressing cells in the grafted spleen. Tissue sections were stained with the indicated antibody combinations. Higher magnification images (lower panels) are indicated by an inserted rectangle in the upper images. Scale bars indicate 100 μm and 50 μm in upper and lower panels, respectively. (n = 3).

To further ascertain whether Tlx1-expressing cells in the spleen function as mesenchymal progenitor cells *in vivo*, we carried out lineage tracing of Tlx1-expressing cells in the neonatal spleen by administrating tamoxifen to *Tlx1*^*CreER-Venus*^; *Rosa26-tdTomato* mice (P7) to mark Tlx1-expressing cells and their progeny. Seven weeks after the final tamoxifen treatment, we examined the contribution of neonatal Tlx1-expressing cells to the mature stromal compartments in the spleen. As shown in Fig. 5, tdTomato-positive cells were mainly observed in the RP; however, a small number were also detectable in the WP. These tdTomato^+^ cells included MAdCAM-1^+^ MRCs lining the marginal sinus that surrounds the WP (Fig. 5a), podoplanin^+^ FRCs localized adjacent to the central arterioles (Fig. 5b) and CD35^+^CXCL13^+^ FDCs (Fig. 5c), indicating that neonatal Tlx1-expressing cells have the potential to differentiate into all mature stromal cells in the spleen. The tdTomato signal appears to be present in the processes of the FRC, but not of the FDCs, which is likely due to the large size of tdTomato protein dimer that prevents its access to the fine FDC processes. Furthermore, when we examined the differentiation potential of Tlx1-expressing cells in the young adult spleen (P28) by tamoxifen-mediated labeling of their progeny 7 weeks after the treatment, Tlx1-expressing cells were found to retain the potential to give rise to MRCs, FRCs, and FDCs (Fig. 5d–f). To confirm that these mature stromal cells in the spleen are derived from bona fide spleen-resident Tlx1-expressing cells, we transplanted tamoxifen-treated embryonic spleen (E17.5) from *Tlx1*^*CreER-Venus*^; *Rosa26*^*tdTomato*^ mice under the kidney capsule of *Tlx1*^*+/+*^ wild-type mice and examined the differentiation of Tlx1-expressing cells in the ectopically developed spleen graft 4 weeks later (Fig. 6a). As shown in Fig. 6b–d, we were able to detect tdTomato^+^ cells among FDCs, FRCs, and MRCs in the grafted spleen, indicating that these cells originated from Tlx1-expressing cells of the spleen proper. Taken together, these findings indicate that Tlx1-expressing cells in the neonatal and young adult spleen are mesenchymal progenitor cells with the potential to differentiate into all of the mature stromal cells in the spleen.

### LTβR- and TNFR-mediated signaling promotes differentiation of Tlx1-expressing cells into mature stromal cells

**Figure 7:**
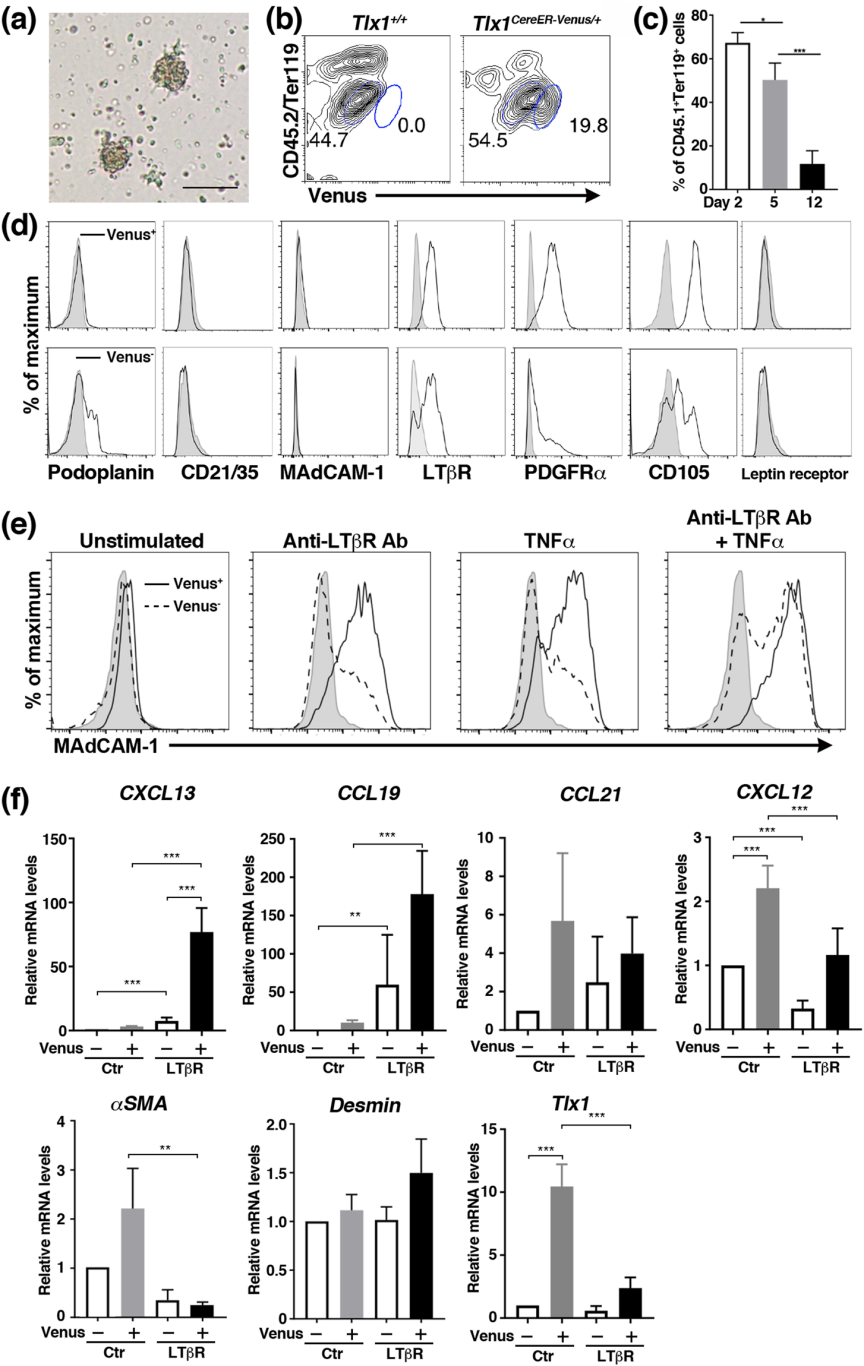
Stimulation of the LTβR and/or TNFR induces differentiation of Tlx1-expressing cells under 3D culture conditions. (**a)** Representative bright-field image showing spleen stromal cell aggregates formed in a Matrigel-embedded 3D culture at day 10. Scale bar indicates 150 μm. (n = 4). (**b**) Representative flow cytometric profiles of Venus^+^ and Venus^−^ stromal cell populations maintained in culture at day 10. Tlx1 expression was defined by Venus fluorescence intensity of CD45.2^−^Ter119^−^ cells from the spleen of *Tlx1*^*CreER-Venus/+*^ mice (P7), as compared with that of CD45.2^−^Ter119^−^ cells from the spleen of *Tlx1*^*+/+*^ mice (P7) under the same culture conditions. Gates used to identify the Venus^+^ cell population are outlined by blue ellipses and numbers adjacent to the outlined areas indicate percent events in each gate. (**c**) Reduction in the frequency of CD45.2^+^ Ter119^+^ hematopoietic cells during the 3D culture. Bars indicate the percentage of CD45.2^+^ Ter119^+^ cells among total cells in the culture. *P < 0.05, ***p < 0.001 (n = 3) (**d**) Representative flow cytometric histograms showing surface marker expression on cultured Venus^+^ and Venus^−^ cells from the spleen of *Tlx1*^*CreER-Venus*^ mice. (n = 3). (**e**) Representative flow cytometric histograms showing expression of MAdCAM-1 on cultured splenic Venus^+^ and Venus^−^ cells stimulated with the anti-LTβR antibody, TNF*α* or both. Solid and dotted lines indicate fluorescence intensity of Venus^+^ and Venus^−^ cells, respectively. Shaded histograms indicate unstimulated controls. (n = 3). (**f**) Expression of mRNA o**f** genes encoding chemokines and mesenchymal cell markers in cultured Venus^+^ and Venus^−^ cells unstimulated or stimulated with the anti-LTβR antibody for 48 hours. Data were normalized to β*-actin* and the level of mRNA transcripts in unstimulated Venus^−^ cells was arbitrarily set to 1. **P < 0.05, ***p < 0.01 (n = 3).

Given that both LTβR and TNFR signals, primarily provided by B cells, have been demonstrated to participate in the correct architectural organization of the WP in the spleen^15,16^, we lastly assessed the potential of Tlx1-expressing cells as lymphoid tissue organizer cells, responsive to LT and TNF signals, to differentiate into mature stromal cells by using a novel 3D culture system with Matrigel-embedded splenic stromal cells. In contrast to the rapid disappearance of Venus fluorescence of splenic stromal cells in conventional two-dimensional cultures (data not shown), this 3D culture system enabled maintenance of Venus^+^ cells as cell aggregates that also contained Venus^−^ cells (Fig. 7a,b**)**, whereas CD45^+^ Ter119^+^ hematopoietic cells were gradually lost during culture (Fig. 7c). The Venus^+^ cells isolated from the cell aggregates maintained an *in vitro* phenotype similar to that seen *in vivo*, characterized by expression of mesenchymal progenitor cell markers, including CD105, PDGFR*α* and LTβR, but with lack of expression of the leptin receptor as well as mature splenic stromal cell markers, such as podoplanin and CD21/35 (Fig. 7d**)**. One exception was MAdCAM-1, which was not expressed by the cultured Venus^+^ cells, probably owing to the lack of environmental signals *in vitro*. In contrast, the Venus^−^ cells appeared to be a heterogeneous cell population in terms of their expression levels of podoplanin, CD105, PDGFR*α* and LTβR (Fig. 7d). These 3D culture conditions that maintained the Tlx1^+^ cells allowed us to perform mechanistic studies. We added an LTβR agonistic antibody and/or TNF*α* into the cultures and then 48 hours later evaluated the expression of MAdCAM-1 as a marker of differentiation into MRCs. MAdCAM-1 expression was uniformly induced on Venus^+^ cells by both anti-LTβR agonistic antibody and TNF*α*, and it was further augmented in the presence of both of these stimuli (Fig. 7e). Furthermore, gene expression analyses revealed that LTβR-stimulation significantly upregulated genes encoding CXCL13 and CCL19, which are abundantly expressed by FDCs and FRCs, respectively, in Venus^+^ cells, as compared to unstimulated controls (Fig. 7f). Conversely, the expression of *CXCL12*, a hallmark of Tlx1-expressing cells among the splenic stromal cells^23^, and *αSMA*, expressed by perivascular mural cells surrounding the central arterioles, were significantly suppressed after the stimulation (Fig. 7f). This was accompanied by a reduction in *Tlx1* expression itself. There were no significant changes in expression of genes encoding CCL21, another protein expressed by FRCs, or desmin, a pan-mesenchymal cell marker, upon LTβR stimulation (Fig. 7f). In addition, it is worth noting that a fraction of the Venus^−^ cells also expressed MAdCAM-1 after LTβR-stimulation (Fig. 7e), and the expression patterns of chemokine genes in Venus^−^ cells were changed upon LTβR-stimulation, similar to the changes observed in Venus^+^ cells (Fig. 7f).

**Figure 8:**
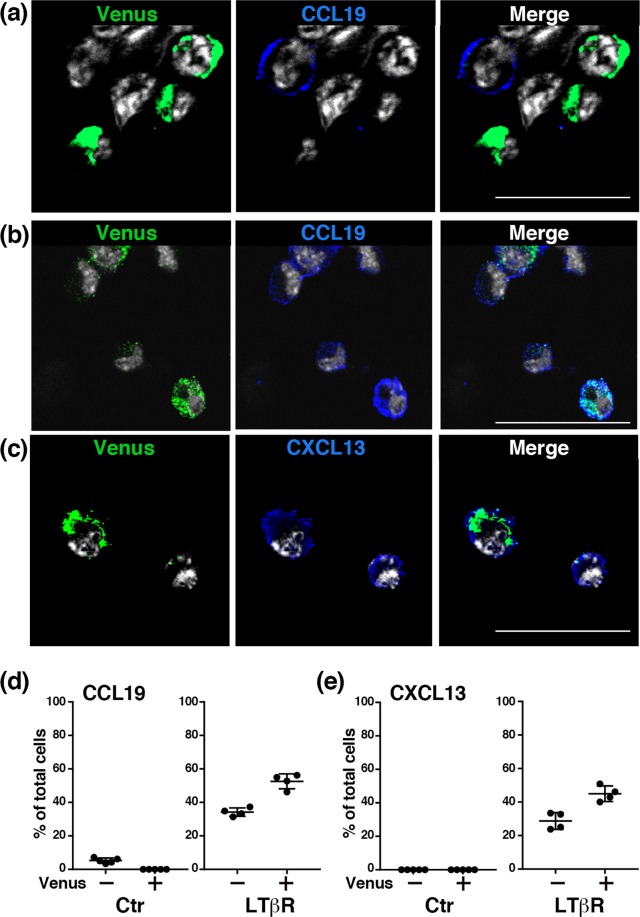
Expression of CCL19 and CXCL13 proteins by 3D-cultured spleen cells upon LTβR stimulation. (**a**) Representative images of Venus and CCL19 expression by cells from the spleen of *Tlx1*^*CreER-Venus*^ mice (P7) under the unstimulated condition. Scale bars indicate 30 μm. **(b)** Representative images of Venus and CCL19 expression by cells after LTβR stimulation for 48 hours. **(c)** Representative images of Venus and CXCL13 expression by cells after LTβR stimulation. **(d)** Frequencies of CCL19^+^ cells in Venus^+^ and Venus^−^ cell populations under unstimulated (650 cells from 5 independent experiments) and stimulated (366 cells from 4 independent experiments) conditions. (mean ± SD). **(e)** Frequencies of CXCL13^+^ cells in Venus^+^ and Venus^−^ cell populations under unstimulated (722 cells from 5 independent experiments) and stimulated (514 cells from 4 independent experiments) conditions. (mean ± SD).

To further evaluate chemokine protein expression at the single cell level, we carried out immunofluorescence staining of cultured cells under LTβR-stimulated and unstimulated conditions by using the anti-Venus antibody in combination with an anti- CXCL13 or CCL19 antibody. Under unstimulated conditions, about 6% of Venus^−^ cells were positive for CCL19, whereas neither CCL19 nor CXCL13 were detectable in Venus^+^ cells (Fig. 8a,d,e). However, upon LTβR-stimulation, the frequencies of CCL19^+^ and CXCL13^+^ cells dramatically increased to 52% and 45% in the Venus^+^ cell population and 34% and 29% in the Venus^−^ cell population, respectively (Fig. 8b-e). Since the intensity of Venus expression in individual cells appeared to be profoundly reduced and varied upon LTβR-stimulation (Fig. 8b,c), the Venus^−^ cell population might also include Venus^+^ cells that lost Venus expression as a result of LTβR-mediated stimuli. Thus, these findings indicate that Tlx1-expressing cells have the ability to differentiate into MRCs, FDCs and FRCs when provided LTβR- and TNF-signals, supporting their potential role as lymphoid tissue organizer cells in the neonatal spleen.

## Discussion

We demonstrate here by genetic lineage tracing that postnatal mesenchymal cells expressing Tlx1, one of the embryonic spleen mesenchyme transcription factors required for proper spleen organogenesis, have the potential to differentiate into mature splenic stromal cells, including FRCs, FDCs and MRCs. Using a 3D culture system, we also showed that these cells behave like LTo cells and/or their precursors in terms of their responses to LTβR and TNFR-mediated signals.

In neonates, Tlx1^+^ cells are selectively found in the spleen, where they are mainly localized outside of the WP area, surrounding the MRCs; *α*SMA^+^ mural cells surrounding the central arteriole also express Tlx1. This distribution pattern in the spleen resembles mesenchymal cells that express Tcf21, a transcription factor that is also expressed in the embryonic spleen primordium and is required for spleen organogenesis^29^. Tlx1^+^ and Tcf21^+^ cells in the postnatal spleen also show phenotypic and functional similarities. Both cell types express mesenchymal progenitor markers such as PDGFRβ, similar to those in the bone marrow, but do not express the leptin receptor, a clear distinction from the bone marrow progenitors^30^. Among the spleen mesenchymal cells, both Tlx1^+^ and a subset of Tcf21^+^ cells are the main source of CXCL12^30^, a key niche factor supporting hematopoietic stem cell (HSC) survival. Although the *in vitro* and *in vivo* differentiation potential of Tcf21^+^ cells remains unclear, Tlx1^+^ cells, which in part functionally overlap Tcf21^+^ cells, appear to be the splenic equivalent of mesenchymal progenitors serving as the HSC niche, just as mesenchymal progenitors constitute the major cell population of the HSC niche in the bone marrow^30^. Furthermore, considering that the descendants of Nkx2-5^+^ mesenchymal cells in the postnatal spleen retain LTo-like activity to regenerate the splenic microenvironment following resolution of a viral infection^19^, mesenchymal cells derived from embryonic splenic mesenchyme that maintain expression of transcription factors involved in spleen organogenesis appear to be the mesenchymal progenitor cells of the postnatal spleen. This notion is also supported by the observation that the diversity and positional identity of mesenchymal progenitors in different organs reflect their selective expression of key transcription factors that are involved in organogenesis^27^. In addition, when compared with neonates, adult Tlx1^+^ cells appear to lose their potential to become mature splenic stromal cells, which correlates well with the decreased level of Tlx1 expression in these cells. However, adult Tlx1^+^ cells might still regain the potential to differentiate into mature splenic stromal cells under conditions of tissue repair when there is massive cell death of mature spleen stromal cells, e.g., after severe virus infections and inflammation. In support of this possibility, we have previously demonstrated that LPS treatment, which mimics systemic bacterial infection, significantly enhances the level of Tlx1 expression in Tlx1^+^ cells^23^. An alternative, but not mutually exclusive, possibility is that mesenchymal LTo-like cells, distinct from mesenchymal progenitors derived from embryonic spleen mesenchyme, also contribute to maintain the structural integrity of the adult spleen microenvironment. For example, FDC precursors have been reported to exist in non-lymphoid organs, including adipose tissues, as PDGFRβ^+^ perivascular cells with the potential to express MAdCAM-1 and CXCL13 in response to LTβR and TNFR signals^31^.

As candidate splenic LTo cells, Tlx1^+^ cells express a high level of LTβR and have the capacity to respond to LTβR- and TNFR-mediated signals, which leads to the expression of MAdCAM-1, a characteristic marker for marginal sinus-lining MRCs. They also respond to express chemokines CXCL19 and CXCL13 secreted by FRCs and FDCs, respectively, that support T and B cell positioning in the WP. Under physiological conditions, the structural maturation of the WP and surrounding marginal zone, including separation of T and B cell areas and marginal sinus formation, occurs postnatally via the interaction of LTo cells with LTi cells^15–18^, although the regional separation of the WP and the RP starts before birth^32^. B cells have been demonstrated to be a major LTi cell that interacts with LTβR-expressing LTo cells by providing their ligand, LT*α*1β2, for WP formation in the neonatal spleen^15,16^. In addition to LTα1β2, TNFα, probably expressed by B cells, has also shown to be involved in splenic WP formation, especially for correct marginal sinus formation^33^, although the effect of TNFα on WP formation is restricted to the spleen, not observed in lymph nodes, and is milder than that of LT*α*1β2^18,33,34^. For instance, the loss of TNFα causes a reduction in podoplanin and CCL21 expression by FRCs as well as MAdCAM-1 expression in the marginal sinus of the spleen, but there is no effect on FRCs and MRCs in the lymph nodes^18,33^. In line with this, the expression of the MRC marker MAdCAM-1 on Tlx1^+^ cells is induced either by LTβR- or TNFR-mediated signaling, but is synergistically up-regulated in the presence of both signals, indicating that both LTβR and TNFR signals are required for the complete differentiation of MRCs. Detailed changes in the distribution of chemokine-expressing LTβR cells in the neonatal (~P5.5) spleen have recently been reported in the context of the spatial organization of T and B cells^15^. Three types of mesenchymal cells were identified: desmin^+^ *α*SMA^+^ cells expressing CCL19 and CCL21 in the inner zone of the WP, colocalized with T cells; desmin^+^ *α*SMA^−^ cells expressing CXCL13 in the outer zone of the WP, colocalized with B cells, and desmin^high^ *α*SMA^−^ cells lacking any chemokine expression at the outermost WP^15^. Since *αSMA* and *desmin* genes are expressed in Tlx1^+^ cells maintained in the 3D cultures, and LTβR-stimulation reduces expression of *αSMA*, but not *desmin*, in these cells (Fig. 7f), it appears that Tlx1^+^ cells represent the desmin^+^ *α*SMA^+^ cells localized to the inner zone of the WP as well as being the precursors of desmin^high^ *α*SMA^−^ cells at the outermost WP. This model is also supported by our immunohistochemical data on the localization of Tlx1^+^ cells in P7 spleen.

A two LTo-cell model is currently proposed for lymph node formation^35^; vascular endothelial LTo cells for initiating recruitment of lymphocytes to the correct site, and mesenchymal LTo cells for subsequent structural compartmentalization. In this regard, CD31^+^CD105^+^CD201^+^MAdCAM-1^+^ endothelial-like LTo cells present in the neonatal spleen have been reported to be required for ectopic regeneration of the spleen^36^. These cells express high levels of *LT*β*R* and *TNFR1* mRNAs, but lack expression of *Tlx1* as well as *Nkx2.5*^36^, in addition to mesenchymal marker genes, including *PDGFR*β and *NG2*, indicating that they are a subset of LTo cells distinct from the Tlx1^+^ cells described in the present study. Furthermore, the ectopic transplantation of embryonic Nkx2.5^+^ cells into the kidney capsule, a similar experimental approach to identify endothelial-like LTo cells in the neonatal spleen, has been demonstrated to result in the regeneration of T and B cell clusters, but donor-derived cells expressing markers for FRCs, FDCs and MRCs were not detectable^9^. These observations further support the distinction of these endothelial-like LTo cells from embryonic spleen primordium-derived mesenchymal cells that support spleen organogenesis under the transcriptional network of the Nkx2.5-dependent and Tlx1-dependent pathways, both of which are downstream transcriptional targets of Pbx1/2^37,38^. Although we have not carried out transplantation experiments with Tlx1^+^ cells isolated from the neonatal spleen, our phenotypic analyses *in vivo* and differentiation analyses *in vitro* also revealed the possible existence of Tlx1^−^ LTo cells with MAdCAM-1 and/or LTβR expression (Figs. 3 and 7d–f). Further comprehensive investigation of LTo cells in the neonatal spleen by using the 3D culture system that we have established in the present study, in combination with *in vivo* lineage tracing and transplantation analyses, will be required to distinguish the origin and functional relationship between endothelial-like LTo cells and mesenchymal LTo cells, including Tlx1^+^ cells selectively present in the spleen as well as periaortic adventitial and mural PDGFRβ^+^ cells recruited from outside of the spleen^31,39^.

Finally, detailed characterization of the origin and function of stromal cell populations in the neonatal spleen will not only facilitate our understanding of the molecular mechanisms underlying the functional structural organization of the spleen, but also offers a potential clinical target to enhance and regenerate functions of the splenic microenvironment that support immune responses against infections.

## Methods

### Mice and *in vivo* treatments

Mice were housed in a specific pathogen-free facility and all animal experiments were carried out in accordance with approved protocols of the Tokyo University of Science Animal Care and Use Committee. *Tlx1*^*CreER-Venus/+*^ mice have been described previously^22^. *Rosa26*^*tdTomato*^ mice (purchased from The Jackson Laboratories) were maintained on a C57BL/6 background. Tamoxifen (0.1 g/kg body weight; Sigma-Aldrich, St Louis, MO) was delivered by intragastric gavage to induce activation of CreER. Transplantation of the embryonic spleen under the kidney capsule of adult mice was carried out as previously described^40^. Unless otherwise indicated, littermates were used as controls; genotypes and treatments are indicated in the text or figure legends.

### Cell preparations

For stromal cell preparation, a whole spleen was transferred into 100 μl of RPMI-1640 medium (Wako Pure Chemical Industries) and was cut and minced using scissors. After adding 900 μl of RPMI and vortexing, the spleen fragments were allowed to sediment for 1 min and the supernatant was transferred to another tube on ice. This process was repeated, collecting the supernatant each time. After collecting the hematopoietic cells, spleen capsules were digested in RPMI-1640 medium containing 4.0 mg/ml collagenase (Wako Pure Chemical Industries) with 0.1 mg/ml DNaseI (Sigma-Aldrich), and incubated for 20 min at 37 °C, with tapping every 10 min. The supernatant was transferred to another tube containing 3 ml of phosphate buffer containing 2% fetal bovine serum (FBS), 2 mM EDTA and 0.1% sodium azide on ice. This process was repeated twice, collecting the supernatant each time. The residual sediments were homogenized by using a 1 ml syringe fitted with a 27-gauge needle (Terumo). The resultant cell suspension was collected by centrifugation, as previously described^23^.

### 3D Matrigel-embedded cell culture and *in vitro* treatments

For three-dimensional culture, splenic stromal cells (1 × 10^6^) from *Tlx1*^*CreER-Venus/+*^ mice (P7) were pelleted and resuspended into 50 μl drops of Matrigel (BD Bioscience), and plated in the middle of the well of a 24-well plate, which was then inverted to polymerize at 37 °C for 15 min, entrapping the cells in the drop. The plate was then placed upright and then 500 μl of Endothelial Cell Basal Medium-2 (EGM-2), supplemented with EGM-2 MV SingleQuots (Lonza), was added and incubated at 37 °C with 5% CO_2_. The cultures were maintained with medium change every 3 days. Ten days after starting the cell cultures, a LTβR agonistic antibody (2 μg/ml; clone 5G11, abcam) and/or TNF*α* (5 ng/ml; R&D systems) were added to the culture for 48 hours, and the resulting cell aggregates were recovered by mechanical crushing of the Matrigel in phosphate-buffer saline, and then incubating with TrypLE™ Express (Gibco) at 37 °C for 15 minutes with vortexing every 3 min to yield single cells for flow cytometry and immunocytochemistry analyses.

### Flow cytometry and cell sorting

Single cell suspensions from the indicated organs were stained with the indicated antibodies and data were collected on FACSCalibur or FACSCanto^TM^II flow cytometers (BD Bioscience) and analyzed using FlowJo software (TreeStar, Ashland, OR). A FACSArea^TM^II (BD Bioscience) was used for cell sorting. Antibodies used for flow cytometry and sorting are listed in Table S1.

### CFU-F assay and tri-lineage differentiation cultures

The colony-forming unit fibroblast (CFU-F) assay was performed using whole spleen stromal cells prepared from tamoxifen-treated mice (P7) as described above. Cells were plated at a clonal density (1.0 × 10^5^/9 cm^2^) and, after 14 days of culture, colonies were counted under a fluorescent stereomicroscope (Olympus MVX10) with a DP73 digital camera (Olympus, Tokyo, Japan). Differentiation cultures to induce adipocytes, osteocytes and chondrocytes were carried out as previously described^27^.

### Immunohistochemistry and immunocytochemistry

Freshly dissected spleen was fixed in 4% paraformaldehyde overnight followed by incubation gradually in 10%, 20% and then 30% sucrose in PBS. Frozen spleens embedded in OTC compound (Sakura Finetek Japan) were serially sectioned (8 μm) with a cryostat (Leica Camera). Sections were blocked, stained with antibodies and mounted with anti-fade prolong gold (Thermo Fisher Scientific). For immunocytochemistry, single cell suspensions (5 μl in FBS) were spotted onto the center of the circle surrounded by PAP pen Super-Liquid Blocker (Cosmo bio) on the glass slide, air-dried and then were fixed with cold methanol for 10 minutes, followed by cold acetone for 1 minute. After blocking with phosphate buffer containing 3% bovine serum albumin, samples were stained with the indicated antibodies as described for immunohistochemistry. Images were acquired with either a BIOREVO BZ9000 (KEYENCE) or a confocal laser-scanning microscope (FV1000-D, Olympus Corporation). Staining antibodies are listed in Table S2.

### qPCR and RT-PCR

Total RNA was extracted with a ReliaPrep RNA cell Miniprep System (Promega Corporation), according to the manufacturer’s instructions. The cDNAs were synthesized using the Superscript VILO cDNA synthesis system (Thermo Fisher Scientific). Real-time PCR was performed using SYBER Premix Ex Taq (Takara) and the CFX384 Real-time System (Bio-Rad). RT-PCR was carried out, as previously described^41^. The PCR primer sequences are listed in Table S3.

### Statistical analysis

The numbers of mice used for each experiment are shown in the Figure legends. Differences between groups were evaluated using Prism 7 software (GraphPad Software, La Jolla, CA). Values are expressed as mean ± standard deviation unless otherwise indicated, and statistical significances were compared with 2-tailed Student’s t test for 2 groups, one-way ANOVA with Dunnet’s test for multiple comparison. A *P* value of less than 0.05 was considered significant.

## Supplementary information

Supplementary Information.

## Data Availability

The authors declare that all other data supporting the findings of this study are available within the article and its Supplementary Information Files.
