# Supplementary material for: Transcription factor Tlx1 marks a subset of lymphoid tissue organizer-like mesenchymal progenitor cells in the neonatal spleen
Source: Sci Rep. 2019 Dec 31;9:20408. doi: 10.1038/s41598-019-56984-w (PMC6938487; doi:10.1038/s41598-019-56984-w)
Supplement: Supplementary file 1 — Supplementary Information. [file 41598_2019_56984_MOESM1_ESM.pdf]

Transcription factor Tlx1 marks a subset of lymphoid tissue organizer-like mesenchymal progenitor cells in the neonatal spleen

Yuta Ueno[1, 2], Keiko Fujisaki[1, 2], Shoko Hosoda[1, 2], Yusuke Amemiya[1, 2], Shogo Okazaki[1], Chihiro Notsu[1], Chiharu Nishiyama[2], Yo Mabuchi[3], Yumi Matsuzaki[4], Akihisa Oda[1,5] and Ryo Goitsuka[1, 6, *]

[1]Division of Development and Aging, Research Institute for Biomedical Sciences, Tokyo University of Science, Chiba, Japan

[2]Laboratory of Molecular Biology and Immunology, Department of Biological Science and Technology, Tokyo University of Science, Tokyo, Japan

[3]Department of Biochemistry and Biophysics, Graduate School of Medical and Dental Sciences, Tokyo Medical and Dental University, Tokyo, Japan

[4]Department of Life Sciences, Faculty of Medicine, Shimane University, Shimane, Japan

[5]Department of Pediatrics, Nara Medical University, Nara, Japan

[6]Imaging Frontier Center, Tokyo University of Science, Chiba, Japan

Corresponding author:

Ryo Goitsuka, Ph.D., D.V.M., Division of Development and Aging, Research Institute for Biomedical Sciences, Tokyo University of Science, 2669 Yamazaki, Noda, Chiba 278-0022, Japan; e-mail: ryogoi@rs.noda.tus.ac.jp

Supplementary Figure Legends

Fig. S1 Tlx1 is not expressed in the hematopoietic cell compartment of the spleen. Representative flow cytometry gating schemes for the stromal cell compartment and the hematopoietic cell compartment of the spleen from *Tlx1*$^{CreER\text{-}Venus}$ mice (P14).

Fig. S2 Full gel images of Fig. 4d.

Supplementary Figure 1

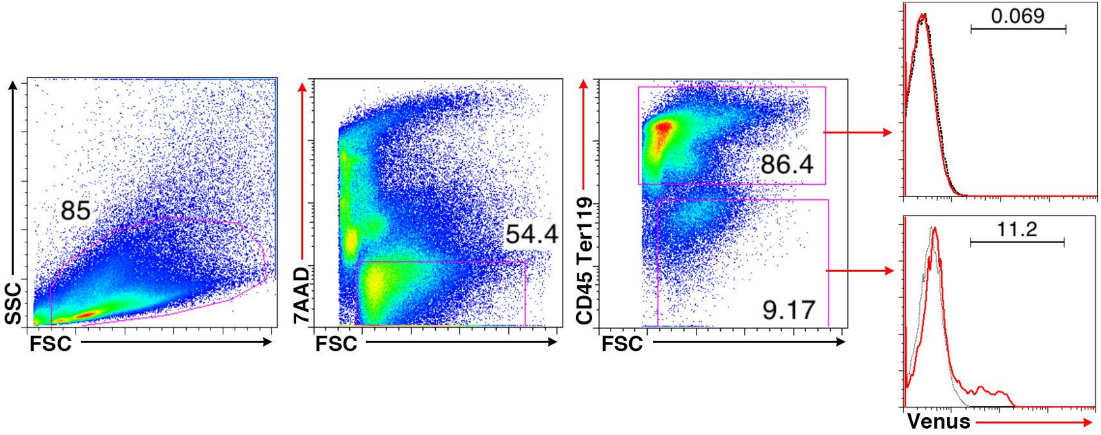

Supplementary Figure 2

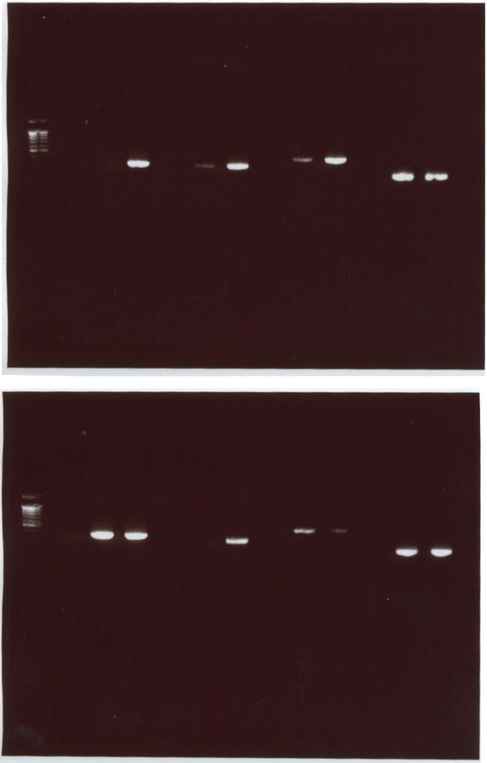

Table S1. Antibodies used for flow cytometry

Antibody	Clone	Conjugated	Company
CD16/32	93	PE	BioLegend
CD21/35	·7E9	FITC	BioLegend
CD21/35	·7E9	APC	BioLegend
CD31	390	Alexa647	BioLegend
CD31	390	PE	BioLegend
CD44	IM7	PE-Cy7	eBioscience
CD45.2	104	PerCP/Cy5.5	BioLegend
CD45.2	104	Brilliant Violet 421	BioLegend
CD105	MJ7/18	Biotin	BioLegend
CD140a	APA5	APC	eBioscience
CD140b	APB5	APC	BioLegend
LeptinR	polyclonal	Biotin	R&D SYSTEM
LtbR	3C8	Biotin	eBioscience
MAdCAM1	MECA367	PE	Santa Cruz Biotechnology
MAdCAM1	MECA367	Biotin	BioLegend
Podoplanin	8.1.1	Biotin	BioLegend
Sca-1	D7	FITC	eBioscience
Ter119	TER-119	APC	BioLegend
Ter119	TER-119	PerCP/Cy5.5	BioLegend
Ter119	TER-119	Pacific Blue	BioLegend
VCAM1	429	Alexa Fluor 647	BioLegend
FDC-M2	FDC-M2	biotin	ImmunoKontact
CD201	eBio1560	PE	BioLegend
Tie2	TEK4	PE	BioLegend
ICAM1	3 E2	Biotin	BD Bioscience
Sca-1	E13-161.7	PE	BD Bioscience
VEGFR2	Avas12	APC	BioLegend
Arm Ham IgG	eBio299Arm	PE-Cy7	eBioscience
Arm Ham IgG	HTK888	Biotin	BioLegend
Syrian Ham IgG	SHG-1	Biotin	BioLegend
Goat IgG	ab37376	Biotin	abcam
Rat IgG2a	17-4321	APC	eBioscience
Rat IgG2a	RTK2758	Biotin	BioLegend
Rat IgG2a	sc-2872	PE	Santa Cruz Biotechnology
Rat IgG1	RTK2071	Biotin	BioLegend
Streptavidin		APC	eBioscience
Streptavidin		eFluor 450	eBioscience

Table S2. Antibodies used for immunohistochemical and immunocytochemical analyses

Antibody	Clone	Conjugated	Company
GFP	Rabbit polyclonal	Purified	MBL
Rabbit IgG	Donkey polyclonal	Alexa Fluor 488	Thermo Fisher
CD3ε	145-2C11	PE	BioLegend
CD3ε	145-2C11	APC	BioLegend
CD3ε	145-2C11	Biotin	BioLegend
B220	RA3-6B2	PE	BioLegend
B220	RA3-6B2	APC	BioLegend
B220	RA3-6B2	Biotin	BioLegend
Podoplanin	8.1.1	Biotin	BioLegend
NG2	Rabbit polyclonal	PE	EMD MILLIPORE
MAdCAM1	MECA367	Biotin	BioLegend
CD21/35	･7E9	FITC	BioLegend
CD21/35	･7E9	Biotin	BioLegend
ERTR7	ER-TR7	Purified	abcam
CD31	390	Alexa647	BioLegend
aSMA	1A4	Cy3	SIGMA
F4/80	BM8	Biotin	BioLegend
CCL19	Goat polyclonal	Purified	R&D SYSTEM
CXCL13	Goat polyclonal	Purified	R&D SYSTEM
CXCL13	Goat polyclonal	Biotin	R&D SYSTEM
Goat IgG	Donkey polyclonal	Alexa Fluor 633	Invitrogen
Streptavidin		Alexa Fluor 488	Thermo Fisher
Streptavidin		Alexa Fluor 548	Thermo Fisher
Streptavidin		Alexa Fluor 633	Thermo Fisher

Table S3. Primers used for qPCR and RT-PCR

β-actin-s	5'-GGCTGTATTCCCCTCCATCG-3'
β-actin-as	5'-CCAGTTGGTAACAATGCCATGT-3'
CXCL13-s	5'-GCTCTGCATCAGTGACGGTA-3'
CXCL13-as	5'-TAATTTCGGGTCAATGCACA-3'
CCL19-s	5'-TCTGCGGGAATCCTGTGACT-3'
CCL19-as	5'-CGGCGACATAGTTGAGGGTTAT-3'
CCL21-s	5'-TCCAAGGGCTGCAAGAGA-3'
CCL21-as	5'-TGAAGTTCGTGGGGGATC-3'
CXCL12-s	5'-GCTCTGCATCAGTGACGGTA-3'
CXCL12-as	5'-TAATTTCGGGTCAATGCACA-3'
αSMA-s	5'-CGTGAAGCCTCACTTCCTACC-3`
αSMA-as	5'-AGAGCCGTTGTCACACACAA-3'
Desmin-s	5' -TGATGAGGCAGATGAGGGAG-3'
Desmin-as	5' -TGAGAGCTGAGAAGGTCTGG-3'
mTlx1 Ex-s	5'-CGCAGATACACAAAGGACAG-3'
mTlx1 Ex-as	5'-TTTCCAGCTCACAGATCTGC-3'
Adipsin-s	5'-ATGGTATGATGTGCAGAGTGTAG-3'
Adipsin-as	5'-CACACATCATGTTAATGGTGAC-3'
PPARγ-s	5'-AACTGCAGGGTGAAACTCTGGGAGATTCTCC-3'
PPARγ-as	5'-GGATTCAGCAACCATTGGGTCAGCTCT-3'
Lpl-s	5'-GAGGACACTTGTCATCTCATTC-3'
Lpl-as	5'-CCTTCTTATTGGTCAGACTTCC-3'
Osteopontin-s	5'-CAGTGATTTGCTTTTGCCTGTTTG-3'
Osteopontin-as	5'-GGTCTCATCAGACTCATCCGAATG-3'
Osteocalcin-s	5'-GACCATCTTTCTGCTCACTCTG-3'
Osteocalcin-as	5'-GTGATACCATAGATGCGTTTGTAG-3'
PTHR-s	5'-GACAAGCTGCTCAAGGAAGTTCTG-3'
PTHR-as	5'-GGAATATCCCACGGTGTAGATCATG-3'
